# Quantitation of cell-free DNA in blood is a potential screening and diagnostic maker of breast cancer: a meta-analysis

**DOI:** 10.18632/oncotarget.21827

**Published:** 2017-10-11

**Authors:** Huadi Wang, Zhen Liu, Jiansheng Xie, Zhanggui Wang, Xiaoyun Zhou, Yong Fang, Hongming Pan, Weidong Han

**Affiliations:** ^1^ Department of Medical Oncology, Sir Run Run Shaw Hospital, College of Medicine, Zhejiang University, Hangzhou, Zhejiang, China; ^2^ Laboratory of Cancer Biology, Institute of Clinical Science, Sir Run Run Shaw Hospital, College of Medicine, Zhejiang University, Hangzhou, Zhejiang, China; ^3^ Department of Radiotherapy, The Second People's Hospital of Anhui Province, Hefei, Anhui, China; ^4^ Department of Medical Oncology, Xiasha Campus, Sir Run Run Shaw Hospital, College of Medicine, Zhejiang University, Hangzhou, Zhejiang, China

**Keywords:** breast cancer, cell-free DNA, screening test, diagnosis

## Abstract

**Introduction:**

Increased cell-free DNA (cfDNA) levels in circulating blood have been associated with higher possibility of breast cancer, however, researchers have not reached an agreement on its analysis.

**Materials and Methods:**

We conducted a meta-analysis of 12 retrospective studies to clarify the value of cfDNA quantification in screening and diagnosis of breast cancer. PubMed, EMBASE, Web of Science and Cochrane library were searched from January, 2000 to October, 2016. Pooled analyses were estimated using a random effects model.

**Results:**

In total, 1003 primary breast cancer patients, 283 cases with benign breast disease and 575 healthy individuals were included. Pooled diagnostic odds ratio (DOR) was 27.63 (95% confidence interval [CI]: 10.96~69.61, I^2^ = 86.2%, *P* < 0.001) in discriminating between breast cancer and healthy controls; the area under the summary receiver operating characteristic (SROC) curve measured 0.91 (95% CI: 0.17~1.00). Analysis of available data in distinguishing breast cancer and benign breast disease showed a pooled DOR of 35.30 (95% CI: 7.58~164.39, I^2^ = 79.9%, *P* = 0.002) with an area under SROC of 0.91 (95% CI: 0.89~0.93). Ethnic group distribution based geographical factors suggested by meta-regression and subgroup analyses explained most of the heterogeneity.

**Conclusions:**

Quantification of cfDNA is a promising test in screening and diagnostic of breast cancer, but population-based standardization of test methods require completion prior to clinical use.

## INTRODUCTION

Breast cancer is the most frequently diagnosed cancer and the leading cause of cancer death among females worldwide [1]. The incidence of breast cancer is still rising, especially in South America, Africa, and Asia [1–2]. Early diagnosis is usually considered to be central to reducing the mortality of breast cancer. Although population-based mammographic screening [3] has contributed to the reduction of death rate of breast cancer in North America and some well-developed European countries, the cost limits its application in developing countries [4]. Therefore, relatively inexpensive tumor biomarkers are also needed.

Circulating cell-free DNA (cfDNA) is the tumor-derived fragmented extracellular DNA which has been detected in human body fluids. As early as 1977, Leon et al. have reported that breast cancer patients contained increased cfDNA in their serum [5]. With advances in knowledge and technology, detection of cfDNA has been applied in prenatal diagnosis [6], disease surveillance, and tumor diagnosis [7]. From cfDNA, we can obtain information regarding cancer, including gene mutations, copy number variation and DNA integrity [8–11]. Quantification of cfDNA has emerged to be a possible tool for early diagnosis of cancers, which has been confirmed in liver cancer and non-small cell lung cancer [12–13]. Numerous clinical studies [14–17] have emphasized that the concentration of cfDNA can be used to distinguish between malignant breast cancer and benign breast nodules. However, there are still inconsistencies in these results and a systematic analyses are required to confirm its diagnostic accuracy. Thus, this meta-analysis was designed to investigate the value of cfDNA quantification as a biomarker for breast cancer and assess the possible factors that influenced the diagnostic efficiency.

## MATERIALS AND METHODS

### Data sources and searches

Four main databases were searched for related studies: PubMed, EMBASE, Web of Science and Cochrane library (from 2000 to October 2016), without language limitations. The combinations of search terms included “breast neoplasms,” “cell-free,” “DNA,” and all of their possible variations. The search strategy was manually adapted according to the citation lists of retrieved articles for sensitivity.

### Study selection

Inclusion criteria consisted of studies in breast cancer and availability of diagnostic data, such as area under the receiver operating characteristic (ROC) curve, specificity and sensitivity. No restrictions on methodology or types of the study were included. Case reports, reviews, conference presentations and duplications were excluded. Two independent reviewers, Z. Liu and H. Wang, evaluated the eligibility of studies. Disagreements were resolved by consensus.

### Data extraction

All the data analyzed were from published papers. Predesigned forms were applied in data collecting. Details listed as follows: first author, year of publication, country, type of study, numbers of cases categorized by age, estrogen receptor (ER), progesterone receptor (PR), human epidermal growth factor receptor 2 (HER-2), tumor stage, and lymph mode metastasis. Sample materials, testing methods, reference genes were collected as well. Diagnostic data was directly extracted from articles or estimated from ROC curves based on the Youden index (sensitivity+specificity-1), as others have published [13, 18]. When nuclear DNA and mitochondria DNA were both measured, the former was used in the analysis.

### Quality assessment

Quality assessment was conducted according to Quality Assessment of Diagnostic Accuracy Studies (QUADAS-2) [19, 20], which is composed of four domains: patient selection, index test, reference standard, and flow and timing. For each domain, the risk of bias and concerns about applicability were evaluated and rated (low risk, high risk, and unclear). The results of the quality assessment were used to investigate potential sources of heterogeneity. Two reviewers scored all the studies independently. Different opinions were discussed until an agreement was reached. If the two reviewers can't reach consensus since the quality of the article was dissatisfying, it would be excluded.

### Statistical analysis

Fourfold tables (tables) for diagnostic test were rebuilt according to the primary publications. Pooled analysis for sensitivity, specificity positive likelihood ratio (PLR), negative likelihood ratio (NLR), and area under the ROC curve (AUC) with their corresponding 95% confidence intervals (95% CI) were calculated using the bivariate random effects model. The diagnostic accuracy for discriminating between breast cancer patients and healthy individuals or patients with benign breast disease were presented as diagnostic odds ratios (DORs) with 95% CI. The statistical heterogeneity was tested through the Q statistic and the variation in OR attributable to heterogeneity was evaluated by statistic. The source of heterogeneity was further investigated using meta-regression and subgroup analyses based on regions, time points of sample collection, sample materials, test methods, and reference genes. The test of publication bias was performed according to the methods described by Deeks et al. [21]. Sensitivity analysis was carried out using leave-one-out method, which is also named influence analysis. All statistical analyses were calculated in STATA v14.0 (Stata Corporation, TX). Statistical significance was defined as *P* < 0.05.

## RESULTS

### Study characteristics and quality assessment

**Figure 1:**
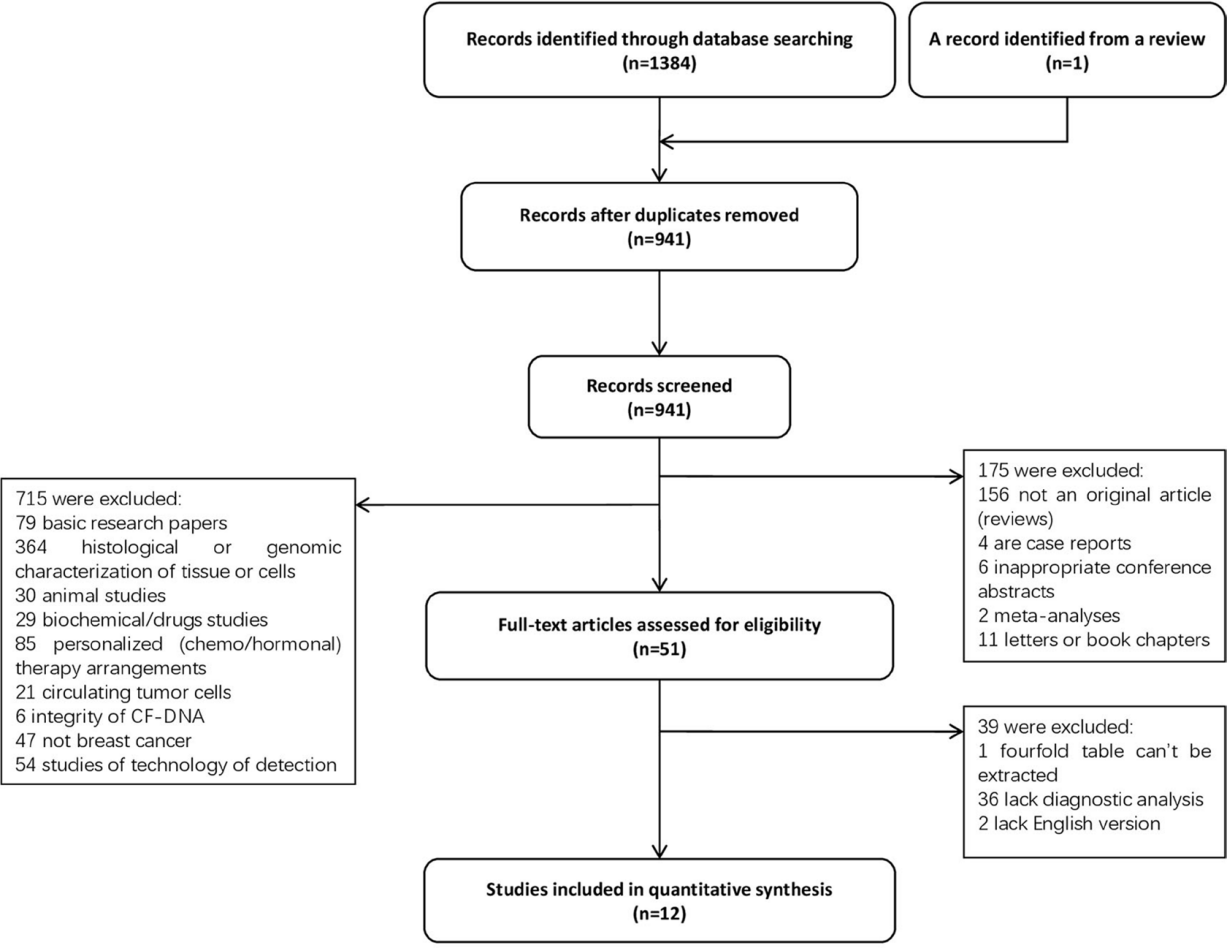
Flow diagram of included studies in this meta-analysis #, the average age of patients who have benign breast disease; $, only parameters of M0 patients are available; @, total numbers of patients in stage I and II; NR, not reported.

A total of 1385 records were retrieved and 12 studies [14–16, 22–30] involving 1807 people met the eligibility criteria (Figure 1). The characteristics of these are included were summarized in Table 1. All the trials were retrospective studies, which involved 8 countries (Portugal, Germany, United Kingdom, Switzerland, Egypt, Israel, China, and Thailand) and 3 regions (Europe, Middle East, and East Asia). Amongst the 12 studies, 1003 primary breast cancer patients and 575 healthy individuals were included; 283 cases with benign breast disease were involved in 8 studies. Eight included studies used quantitative PCR based methods, though they varied in the process of DNA extraction and the choice of reference genes. The other four used fluorescence quantitative analyses. Out of the 12 studies, 8 collected samples of plasma or serum before treatment (surgery or chemotherapy), the others collected samples after surgery. Most breast cancers patients were diagnosed in their fifties and were in stage II~III. Details of reference genes, cut-off values and AUCs in each study were summarized in Table 2. Information of Pre-analytical procedures of cfDNA quantification were listed in Supplementary Table 1.

**Table 1:** Basic characteristics of the included studies

Authors	Year	Country	Number of cases				Age (years)		ER		PR		HER2		Tumor stage				Lymph node metastasis	
			Cancer	Benign	Health	Total	Patients	Control	+	−	+	−	+	−	I	II	III	IV	+	−
**Catarino et al.**	2008	Portugal	175	0	80	255	49	38	NR	NR	NR	NR	NR	NR	34	69	39	7	NR	NR
**Roth et al.**	2011	Germany	63	20	28	111	56	49	41	19	36	24	23	35	25^@^		29	9	42^$^	13^$^
**Hashad et al.**	2012	Egypt	42	30	27	99	NR	NR	22	20	14	28	10	32	7	22	10	3	29	13
**Gong et al.**	2012	China	200	100	100	400	41	44	114	86	NR	NR	NR	NR	9	86	93	13	NR	NR
**Zaher et al.**	2012	Egypt	24	12	30	66	54 49^#^	49.8	NR	NR	NR	NR	NR	NR	NR	NR	NR	NR	NR	NR
**Schwarzenbach et al.**	2011	Germany	102	32	53	187	60 50^#^	50	20	82	36	66	NR	NR	57^@^		44	1	48	54
**Gal et al.**	2004	UK	96	0	24	120	56	39	50	25	NR	NR	NR	NR	NR	NR	NR	NR	49	47
**Huang et al.**	2006	China	61	33	27	121	56 55^#^	54	24	37	NR	NR	NR	NR	11	19	21	10	35	26
**Kohler et al.**	2009	Switzerland	52	26	70	148	64 41^#^	70	NR	NR	NR	NR	NR	NR	NR	NR	NR	NR	NR	NR
**Mahmoud et al.**	2015	Egypt	50	30	20	100	49 44^#^	42.5	34	16	17	33	23	26	17	21	8	4	33	17
**Orathai et al.**	2015	Thailand	100	0	100	200	NR	NR	NR	NR	NR	NR	NR	NR	3	12	17	3	NR	NR
**Agassi et al.**	2015	Israel	38	0	16	54	64	47	33	5	25	13	2	36	18	13	6	0	NR	NR

^#^, average age of patients who have benign breast disease

^$^, only parameters of M0 patients are available

^@^, total number of patients in stage I and II

NR, not reported

**Table 2:** Summary of outcomes of the included studies

Authors	Time of sample collection	Material	Test method	Reference gene	Cutoff value	AUC
**Catarino et al.**	after/before surgery	plasma	real-time qPCR	hTERT	106.0 ng/mL	NR
**Roth et al.**	after surgery before therapy	serum	Quant-iT PicoGreen	/	NR	0.77
**Hashad et al.**	before surgery or therapy	plasma	real-time qPCR	hTERT	34 ng/mL	NR
**Gong et al.**	before surgery or therapy	Serum	real-time qPCR	GAPDH	471 ng/ml	0.93
**Zaher et al.**	before surgery or therapy	serum	Quant-iT PicoGreen	/	600 ng/μL	0.96
**Schwarzenbach et al.**	before surgery	serum	fluorescence-labelled PCR	D13S159 D13S280 D13S282 D10S1765	NR	0.66
**Gal et al.**	before surgery	serum	real-time qPCR	β-globin	NR	0.92
**Huang, et al.**	Before surgery and therapy	plasma	real-time qPCR	β-globin	19 ng/mL	0.95
**Kohler et al.**	Before surgery or therapy	plasma	multiplex real-time qPCR	GAPDH & MTATP 8	1866 GE/mL	0.80
**Mahmoud et al.**	before surgery	plasma	multiplex real time PCR	GAPDH & MTATP 8	2236 copy/μL	0.79
**Orathai et al..**	after/before surgery	plasma	QubitTM fluorometer	/	90 ng/ml	0.96
**Agassi et al.**	before surgery or therapy	serum	fluorescent SYBR Gold stain	/	600 ng/ml	0.83

NR, not reported

Evaluation of the risk of bias and concerns regarding applicability are graphically displayed in Supplementary Table 2. Gal et al. [26] arranged four groups of breast cancer patients, all the other studies reported that the patients were consecutive or random in a certain period of time. All patients included had clear diagnosis with pathological evidence. In general, all studies met the predefined criteria for our review questions and were high in applicability.

### Screening and diagnosis value of cfDNA quantification for BC

**Figure 2:**
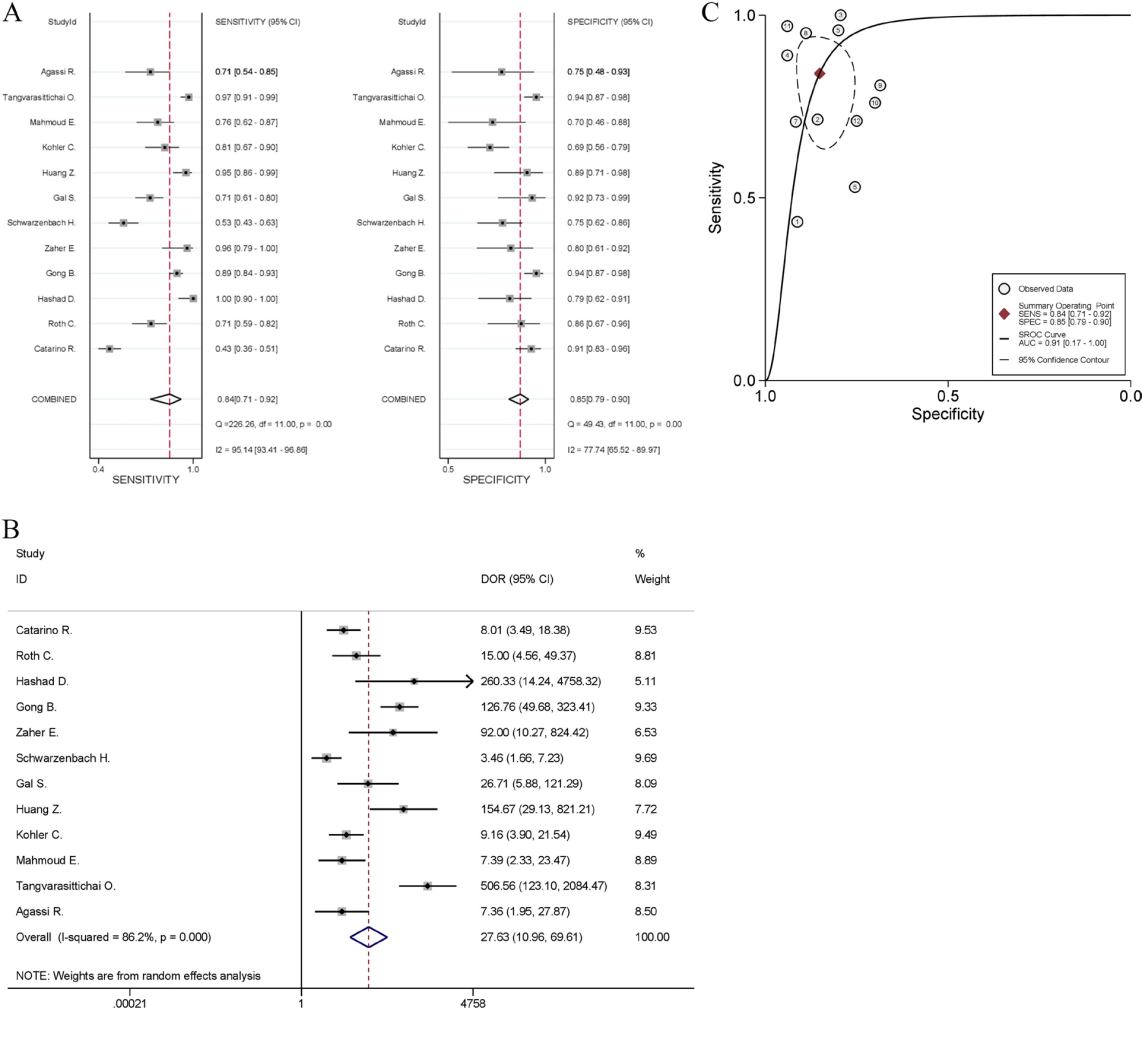
Meta-analysis of the power of cfDNA quantification to discriminate between breast cancer and healthy controls (**A**) Sensitivity and specificity, (**B**) diagnostic odds ratio, and (**C**) summary receiver operating characteristic (ROC) curve. The odds ratios for each trial are represented by squares, and the horizontal line crossing the square represents the 95% confidence interval (CI). The diamonds represent the estimated pooled effect.

All studies reported data for discriminating between breast cancer and healthy controls, and showed that high levels of cfDNA significantly pointed to breast cancer. The scatter plot showed in Supplementary Figure 1 and calculation of Harbord test indicated that no significant publication bias (*P* = 0.56) and no small-study effects (*P* = 0.67) were detected. The pooled sensitivity was 84% [95% CI, 71~92%; *I^2^* = 95.14%, Q = 226.26 (*P* < 0.01)] and specificity was 85% [95% CI, 79~90%; *I^2^* = 77.74%, Q = 49.43 (*P* < 0.01); Figure 2A]. PLR and NLR were 5.7 (95% CI, 3.8–8.5) and 0.19 (95% CI, 0.10–0.36), respectively. The DOR value measured 27.63 (95% CI, 10.96~69.61; *I^2^* = 86.2%, *P* < 0.001; Figure 2B), and estimation of summary ROC (SROC) curve displayed an AUC of 0.91 (95% CI, 0.17~1.00; Figure 2C), indicating a good discriminatory accuracy for breast cancer versus healthy individuals.

**Figure 3:**
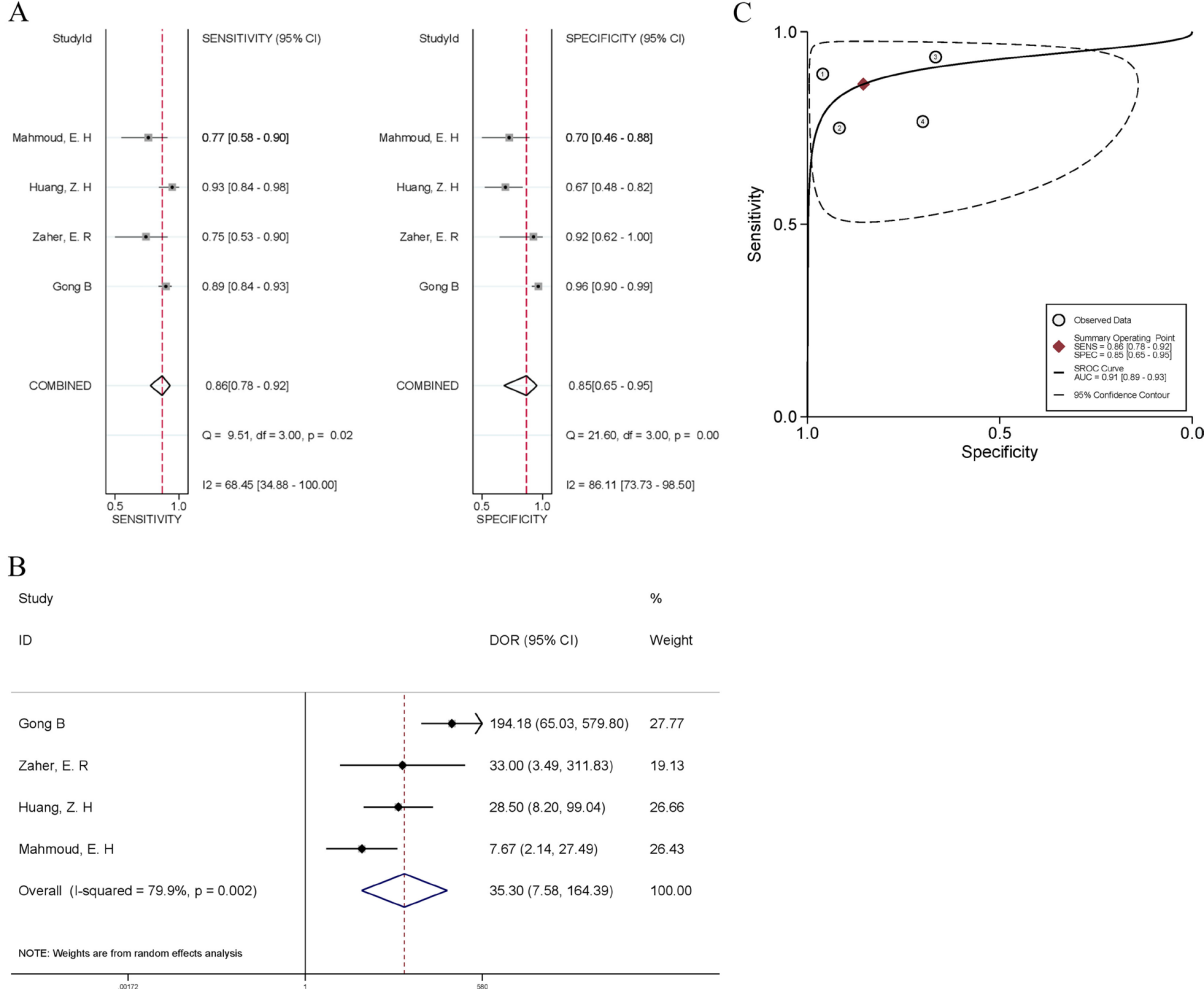
Forest plots of the sensitivity and specificity (**A**), diagnostic odds ratio (**B**), and summary receiver operating characteristic (SROC) curve (**C**) for cfDNA quantification in the discrimination between breast cancer and benign breast disease. The odds ratios for each trial are represented by squares, and the horizontal lime crossing the square represents the 95% confidence interval (CI). The diamonds represent the estimated pooled effect.

Eight studies included patients with benign breast disease, however, only four of them [23–24, 27–28] had diagnostic data available for pooled quantitative analyses. For these four studies, the pooled sensitivity and specificity were 0.86 (95% CI, 78~92%; Q = 9.51, *I^2^* = 68.54%, *P* = 0.02) and 85% (95% CI, 65~95%; Q = 21.60, *I^2^* = 86.11%, *P* < 0.01), respectively (Figure 3A). The pooled DOR was 35.30 (95% CI, 7.58~164.39; Figure 3B), which had significant heterogeneity (*I^2^* = 79.9%, *P* = 0.002). The area under the SROC curve was 0.91 (95% CI, 0.89~0.93; Figure 3C). Both DOR and AUC suggested that the quantification of cfDNA might become a diagnostic tool in differentiating breast cancer from benign lesions.

The concentration of cfDNA was also associated with molecular subtypes of breast cancer and nodal status (Supplementary Table 3). Although cfDNA levels were not associated with ER or PR, they were significantly higher in HER-2-positive patients than in HER-2-negative patients [15, 28]. Most researchers [15, 25–28, 30–31] showed a higher level of cfDNA in node-positive patients compared to node-negative patients, and as more lymph nodes are involved, more cfDNA could be detected in circulation [28]. Also, two studies [15, 30] reported significant differences in the level of cfDNA between node-positive patients and node-negative patients, which suggested that the concentration of cfDNA might be a possible marker of early lymph node metastasis in breast cancer.

### Major clinical heterogeneity sources

To find the source of heterogeneity, firstly, random effects meta-regression analysis was used to assess covariates involved in these studies. Factors including “regions (Europe, Middle East, and East Asia)”, “sample materials (plasma or serum)”, “test methods (polymerase chain reaction (PCR)-based or not)”, “time of sample collection (before or after treatment)”, and “Method of extraction (QIAamp DNA Blood Mini Kit, Qiagen DNA Blood Mini Kit, Nucleic-Spino Plasma XS Kit, and Other)” were included in univariate analysis. The results suggested that “regions” accounted for most of heterogeneity and explained 82.54% between-study variance. Although “test methods” alone was not responsible for the heterogeneity (Coef. = 0.70, 95% CI, -1.64~3.04, *P* = 0.53), in multivariate analysis, “test methods” and “regions” altogether explained 87.09% of the between-study variance. The other factors could not explain the heterogeneity, and details of the calculation were showed in Table 3.

**Table 3:** Meta-regression of effects of study characteristics on diagnostic accuracy of cfDNA quantification

Variable		Regression Coefficient (95% CI)	SE	*P*	I^2^ (95% CI)
Area	Europe	−3.14 (−4.74 to −1.54)	0.71	0.002	54.67%
	Middle East	−2.49 (−4.36 to −0.61)	0.83	0.15	
Sample material	Plasma (vs. serum)	0.61 (−1.61 to 2.83)	0.99	0.55	87.31%
Test method	Not PCR-based (vs. PCR- based)	0.70 (−1.64 to 3.04)	1.05	0.53	86.31%
Time of sample collection	Before (vs. after)	−0.35 (−2.88 to 2.18)	1.14	0.76	87.23%
Method of extraction	Nucleic-Spino Plasma XS Kit	2.22 (−1.73 to 6.17)	1.71	0.23	82.82%
	QIAamp DNA Blood Mini Kit	−0.50 (−3.84 to 2.84)	1.45	0.74	
	Other (except Qiagen DNA extraction MiniKit)	−0.30 (−3.63 to 3.03)	1.44	0.84	
Area & Test Method	Europe	−3.06 (−4.66 to −1.46)	0.69	0.002	52.17%
	Middle East	−2.66 (−4.58 to −0.75)	0.83	0.012	
	Not PCR-based	0.67 (−0.85 to 2.19)	0.66	0.341	

SE, standard error.

**Figure 4:**
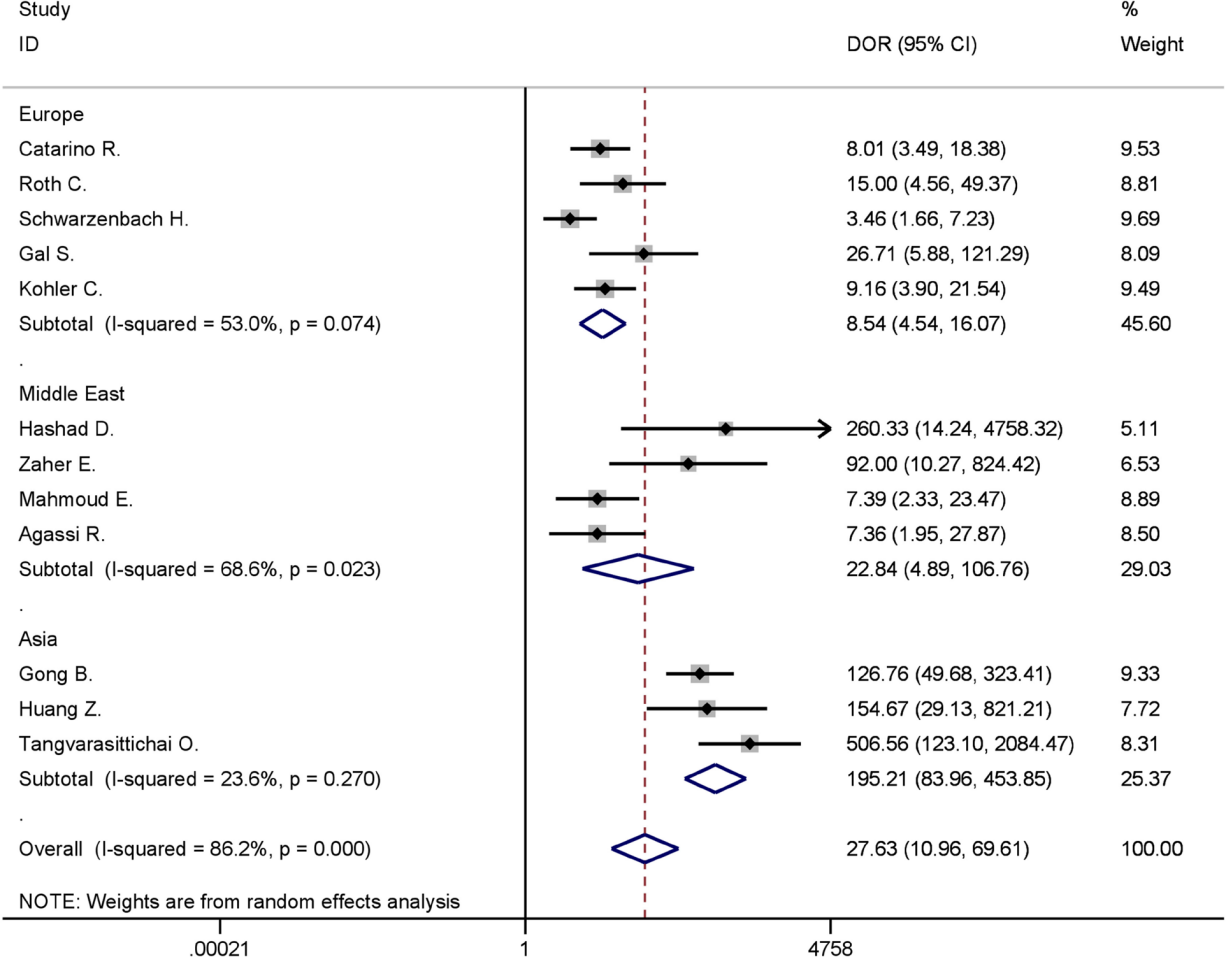
Forest plot for subgroup analysis of different areas in discriminating between breast cancer and healthy controls

The geographical grouping (Europe, Middle East, and East Asia) contained the differences in ethnic and genetic characteristics in these regions, thus, subgroup analysis was used to further evaluate the accuracy of diagnosis test in each region. As shown in Figure 4, the pooled DOR for cfDNA test were 8.54 (95% CI, 4.54–16.07; *I^2^* = 53.0%, *P* = 0.074) in Europe, 22.84 (95% CI, 4.89–106.76; *I^2^* = 68.66%, *P* = 0.023) in Middle East, and 195.21 (95% CI, 83.96–453.85; *I^2^* = 23.6%, *P* = 0.270) in East Asia. The diagnostic performance in East Asia increased dramatically while the heterogeneity went down to a low level. Pooled analysis of European studies showed moderate heterogeneity, however, the DOR did not indicate fairly good discriminatory test performance. Many other factors still needed to be considered and analyzed in studies from Middle East.

Sensitivity analysis, using leave-one-out sensitivity analysis (Supplementary Figure 2) with a random effect model, revealed that the pooled effect estimates would not be influenced by any single study and maintain their stability.

## DISCUSSION

The incidence and mortality of breast cancer in less developed countries are still increasing. Early screening and reliable diagnosis are essential for breast cancer treatment. Various investigations [14–16, 22–34] have demonstrated that the quantification of cfDNA was potentially an effective biomarker for breast cancer diagnosis. In this meta-analysis, quantification of cfDNA as a screening tool for breast cancer had a pooled sensitivity of 84% and a pooled specificity of 85%. The DOR was 27.63 and the AUC value was 0.91, which reached a high level of evaluation criteria and indicated a high degree of overall diagnostic accuracy. The cfDNA level was reported higher in HER-2 or node positive patients but was not associated with ER and PR status. Subgroup and meta-regression analyses found “regions” was the main source of heterogeneity, revealing the heterogeneity of breast cancer among ethnic groups. Additionally, as a diagnostic tool, high level of cfDNA also pointed to a higher risk of breast cancer, with a DOR of 35.30 and an AUC of 0.91, indicating a diagnostic value of cfDNA quantification for breast cancer.

Sources of heterogeneity was evaluated by meta-regression and subgroup analyses. Nearly 90 percent of the heterogeneity could be explained by the mixed effect of “regions” and “test method”, and the former is the main factor. Subgroup analysis provided more details of the ethnicity-based regional grouping. The heterogeneity between three studies from East Asia [23, 27, 29] decreased to a quite low level (*I^2^* < 30.0%), and studies from China [23, 27] had no heterogeneity (*I^2^* = 0%), both of which used real-time quantification PCR. Four studies from Middle East were done in Egypt and Israel, and there remained moderate heterogeneity within this subgroup. The major covariate affecting these trials was “testing method”: Hadshad et al. [15] and Mahmoud et al. [28] quantified cfDNA with PCR-based detection method. Zaher et al. used a fluorescence-based DNA assay kit (PicoGreen), and Agassi et al. chose a fluorescent dye [35] to measure cfDNA directly in the diluted samples. Pre-analytical procedures of cfDNA quantification do add heterogeneity according to the information we collected (Supplementary Table 1), however, only 14.50% of between-study variance was explained by “method of extraction”. Missing information and changing methods hindered further analyses, and excessive confounding factors can also lead to unreliable analysis results. Generally, PCR is regarded as a more sensitive approach in the quantification of cfDNA, however, the standardization of cfDNA quantification methods remains one of the problems confronted in the way of further clinical application. Therefore, we recommend a unified technique in future studies of cfDNA at least in a specific region, in order to guarantee the sensitivity of detection and establish guidelines in this area.

Recently, Lin et al. [36] published a meta-analysis to comprehensively evaluate the cfDNA-based early detection methods for BC. They included literature measuring cfDNA quantification, integrity, methylations, loss of heterogeneity and etc., which resulted in heterogeneity between studies. In this meta-analysis, we only included studies containing DNA quantification results. Measured by the same inclusion criteria, more studies could be included in our study (*n* = 12 vs. *n* = 9). Our comprehensive literature search was supported by a lack of evidence for publication bias through Deeks and Harbord test. Although significant heterogeneity was observed in their study, Lin *et al.* demonstrated that none of the methodological covariates (“country” and “assay methods”) produced major heterogeneity (*P* > 0.05) in meta-regression analysis. This may be caused by the loose inclusion criteria, which introduced too many influencing factors. Moreover, among the data from Kohler *et al.*'s study [16], Lin et al. mistook the results of screening test using mitochondrial DNA (mtDNA) quantification (cut-off: 463282 GE/ml; sensitivity: 53%; specificity: 87%; *P* < 0.001) for distinguishing BC from patients with benign breast diseases.

However, there remain limitations in our meta-analysis. Firstly, due to the restriction of systematic review and meta-analysis, only population-level data could be extracted; more correlation of subtypes defined by ER, PR or HER-2 as well as lymph node metastasis could not be further analyzed. Secondly, there remained about 1need a space: 10% heterogeneity which did not have clear source. At the pre-analytical phase of cfDNA quantification, the methods of cfDNA extraction and the following assessment lacked standard protocols, which may lead to heterogeneity [37].

In conclusion, this meta-analysis suggests that the quantification of cfDNA can be a potential biomarker for accurately discriminating BC patients from healthy individuals. Sensitivity analyses using various criteria to improve the quality of included studies or reduce the systematic errors in the process of calculation did not alter the results substantially, suggesting that the results of our meta-analysis are robust. Combination of cfDNA quantification and other biomarkers maybe a future direction in BC early diagnosis.

## SUPPLEMENTARY MATERIALS FIGURES AND TABLES



## Abbreviations

cfDNA: cell-free DNA
CI: confidence interval
DOR: diagnostic odds ratio
SROC: summary receiver operating characteristic curve
ER: estrogen receptor
PR: progesterone receptor
HER-2: human epidermal growth factor receptor-2
QUADAS-2: Quality Assessment of Diagnostic Accuracy Studies (2nd edition)
PLR: positive likelihood ratio
NLR: negative likelihood ratio
ROC: receiver operating characteristic curve
AUC: area under the ROC curve
