# Supplementary material for: Quantitation of cell-free DNA in blood is a potential screening and diagnostic maker of breast cancer: a meta-analysis
Source: Oncotarget. 2017 Oct 11;8(60):102336–45. doi: 10.18632/oncotarget.21827 (PMC5731959; doi:10.18632/oncotarget.21827)
Supplement: Supplementary file 1 [file oncotarget-08-102336-s001.pdf]

Quantitation of cell-free DNA in blood is a potential screening and diagnostic maker of breast cancer: a meta-analysis

SUPPLEMENTARY MATERIALS

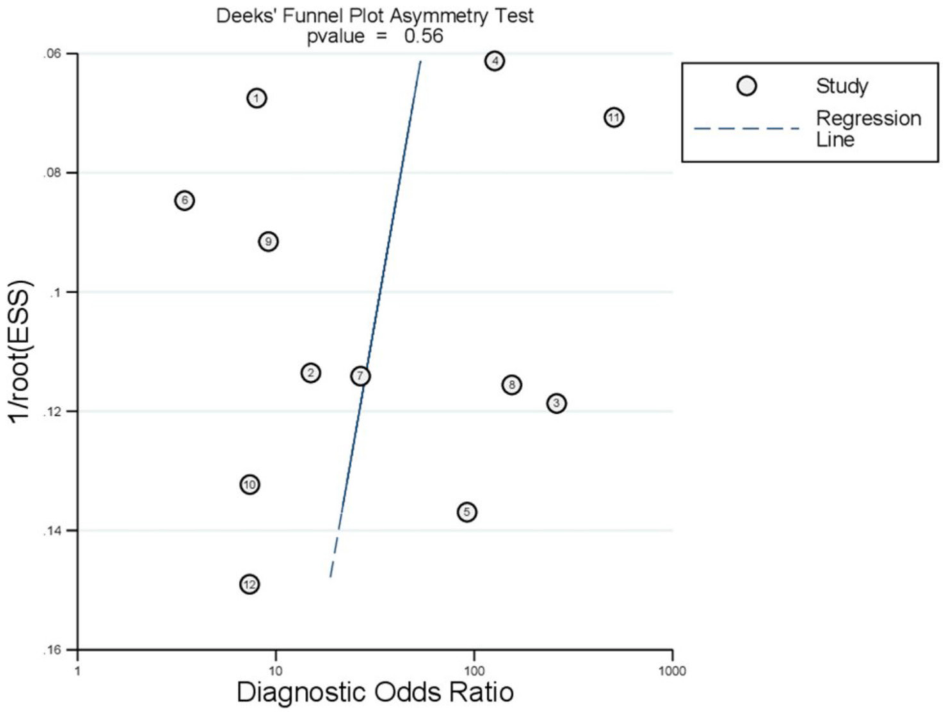

Supplementary Figure 1: Deeks' funnel plot to analyze the likelihood of publication bias.

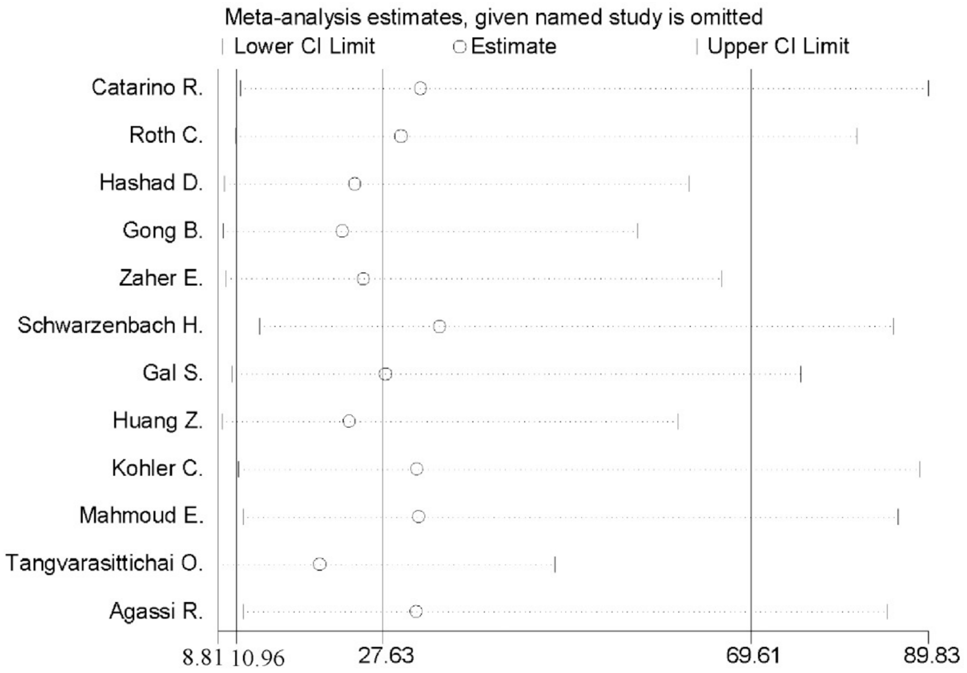

Supplementary Figure 2: Leave-one-out sensitivity analysis using random effect model.

Supplementary Table 1: Pre-analytical phase of cfDNA quantification

Authors	Year	Country	Time between venipuncture and centrifugation	Process of centrifugation method of extraction	Method of extraction	Details of quantification
Catarino et al.	2008	Portugal	Immediately	2500 rpm 10 min	QIAamp DNA Blood Mini Kit (Diagen, Hilden, Germany)	Real-time qPCR; In detail
Roth et al.	2011	Germany	Not mentioned	Not mentioned	Quanti-iT Pico-Green dsDNA Kit (Invitrogen, Karlsruhe, Germany)	Quanti-iT Pico-Green dsDNA Kit
Hashad et al.	2012	Egypt	Not mentioned	1st: 1600 g 10 min<br>2nd: 16000 g 10 min	Qiagen DNA Blood Minikit (Diagen, Hilden, Germany)	Real-time qPCR; In detail
Gong et al.	2012	China	Within 4 hours	2500rpm 5 min	In-house magnetic beads	Real-time qPCR; In detail
Zaher et al.	2012	Egypt	Not mentioned	Not mentioned	Nucleic-Spino Plasma XS Kit (Macherey-Nagel GmbH & Co. KG, Germany)	Quanti-iT Pico-Green dsDNA Kit
Schwarzenbach et al.	2011	Germany	Not mentioned	Not mentioned	QIAamp DNA Blood Mini Kit (Diagen, Hilden, Germany)	Fluorescence-labelled PCR; In detail
Gal et al.	2004	UK	Not mentioned	Not mentioned	QIAamp DNA Blood Mini Kit (Diagen, Hilden, Germany)	Real-time qPCR; In detail
Huang et al.	2006	China	Within 2 hours	1st: 2000 g 10 min<br>2nd: 12000 g 10 min	QIAamp DNA Blood Mini Kit (Diagen, Hilden, Germany)	Real-time qPCR; In detail
Kohler et al.	2009	Switzerland	Immediately	1st: 1600 g 10 min<br>2nd: 16000 g 10 min	High pure PCR template preparation kit (Roche Diagnostics, Germany)	Multiplex real-time qPCR; In detail
Mahmoud et al.	2015	Egypt	Within 2 hours	1st: 3000 g 10 min<br>2nd: 12000 g 10 min	Qiagen DNA extraction Minikit (Diagen, Hilden, Germany)	Nanodrop spectrophotometer & multiplex rt-PCR
Orathai et al.	2015	Thailand	Within 2 hours	1st: 800 g 10 min<br>2nd: 1600 g 10 min	Nucleic-Spino Plasma XS Kit (Macherey-Nagel GmbH & Co. KG, Germany)	QubitTM fluorometer; In detail
Agassi et al.	2015	Israel	Not mentioned	Not mentioned	Nucleic acid fluorochrome SYBR Gold (Invitrogen, Paisley, England)	Do not need extraction and amplification

Supplementary Table 2: Assessment of the methodological quality by the quality assessment of diagnostic accuracy studies tool, version 2 (QUADAS-2)

Study	Risk of Bias				Applicability Concerns		
	Patient Selection	Index Test	Reference Standard	Flow and Timing	Patient Selection	Index Test	Reference Standard
**Catarino et al.**	☺	☹	☺	☺	☺	☺	☺
**Roth et al.**	☺	☹	☺	☹	☺	☺	☺
**Hashad et al.**	☺	☹	☺	☺	☺	☺	☺
**Gong et al.**	☺	☹	☺	☺	☺	☺	☺
**Zaher et al.**	☺	☹	☺	☺	☺	☺	☺
**Schwarzenbach et al.**	☺	☹	☺	☺	☺	☺	☺
**Gal et al.**	☹	☹	☺	☺	☺	☺	☺
**Huang et al.**	☺	☹	☺	☺	☺	☺	☺
**Kohler et al.**	☺	☹	☺	☺	☺	✍	☺
**Mahmoud et al.**	☺	☹	☺	☺	☺	✍	☺
**Orathai et al.**	☺	☹	☺	☺	☺	☺	☺
**Agassi et al.**	☺	☹	☺	☺	☺	☺	☺

☺ = low risk; ☹ = high risk; ✍ = unclear risk

Concerning patient selection bias, despite Catarino et al. included 175 consecutive patients and Gal et al. arranged four groups of breast cancer patients to match their research goal, all the other eight studies reported that the patients were consecutive or random in a certain period of time. Regarding the index test, all studies described the process of measuring the concentration of cfDNA and met the predefined criteria for our review questions. Only Gong et al used a prespecified threshold in their testing cohort, the other studies select the test threshold which optimizes sensitivity and/or specificity. Considering the objectivity of PCR quantification and detailed measurement process provided, whether the laboratory technicians knew patients' diagnoses did not bring any bias in the test results. Since all studies had pathological diagnosis for every patient, there was no concern about reference standard applicability. For the flow and timing domain, Roth et al. took the serum 1~13 years after surgery of the primary tumor, which might have introduced bias due to inappropriate interval between index tests and reference standards. In general, most studies were high in applicability.

Supplementary Table 3: Median concentration of cell-free DNA categorized by molecular features and nodal status

Authors	Healthy	ER			PR			HER2			Lymph node metastasis		
		+	−	*p*	+	−	*p*	+	−	*p*	+	−	*p*
Roth et al.	7641	7868	8351	NR	8124	8149	NR	8149	8100	NR	7517	8149	NR
Hashad et al.	12	204	162	0.124	198	165	0.176	268	167	0.002	289	156	< 0.001
Schwarzenbach et al.	NR	3500	3610	NR	3625	3272	NR	NR	NR	NR	3925	2910	NR
Gal et al.	63	NR	NR	0.15	NR	NR	NR	NR	NR	NR	297[d], 225[e]	195[d],191[e]	0.68
Huang et al.	13	72	62	0.25	NR	NR	NR	NR	NR	NR	69	59	0.38
Mahmoud et al.	90	NR	NR	NR	NR	NR	NR	123027	415	0.0001	705[a], 44595[b], 335792[c]	599	NR
Agassi et al. [#]	395	1076	807	NR	995	1068	NR	NR	NR	NR	1486a	734	< 0.001

[a], N1; [b], N2; [c], N3

[d], relapse-free survival5years; e, relapse-free survival>5years

#, mean concentration

NR, not reported
